# Two species, one island: Retrospective analysis of threatened fauna translocations with divergent outcomes

**DOI:** 10.1371/journal.pone.0253962

**Published:** 2021-07-12

**Authors:** Kelly Rayner, Cheryl A. Lohr, Sean Garretson, Peter Speldewinde

**Affiliations:** 1 Biodiversity and Conservation Science Division, Western Australia Department of Biodiversity, Conservation and Attractions, Perth, Western Australia, Australia; 2 Centre of Excellence in Natural Resource Management, The University of Western Australia, Perth, Western Australia, Australia; Smithsonian Conservation Biology Institute, UNITED STATES

## Abstract

Translocations are globally a popular tool used with the intention of improving threatened species conservation and re-establishing ecosystem function. While practitioners strive for successful outcomes the failure rate of translocations continues to be high. We demonstrate how predictive modelling can contribute to more informed decision making and hence potentially improve the success rate of translocation programs. Two species, the Djoongari (Shark Bay mouse) *Pseudomys fieldi* and the golden bandicoot *Isoodon auratus barrowensis*, were introduced independently to Doole Island in the Exmouth Gulf of Western Australia. We used population viability analysis to critique the outcomes of these translocations and provide an example of how this tool can be incorporated with expert knowledge to predict likely outcomes of translocations. Djoongari did not establish on the island after seven translocations over nine years, while golden bandicoots established a population after just one release event. Retrospective population viability analysis (of data that was unavailable prior to the translocations) predicted and clarified the reasons behind the outcomes of both translocations. Golden bandicoots have considerably higher demographic plasticity than Djoongari, which were never likely to establish on the island. We conclude that the failure of the Djoongari translocation was due to interactions between sparse habitat, native predators and cyclonic storm surges, whereas golden bandicoots have demonstrated habitat flexibility and an ability to recover from multiple natural disasters. As a result we (1) remind conservation planners of the importance of quantifying likely refuges and habitat availability at release sites, (2) suggest practitioners consider how different threats (including natural disasters) may interact at potential release sites and (3) advocate for the incorporation of predictive modelling during the planning stages of translocations, particularly for conservation introductions where no precedent exists for the species’ survival at a particular location.

## Introduction

Translocations are a common management tool used within the field of conservation worldwide [1]. Fauna are moved to improve a species’ conservation status or restore their ecological function to a system where it has been lost [2]. The movement of animals with the goal of creating new populations has been achieved using direct ‘wild to wild’ translocations as well as using captive breeding as an intermediate step to overcome limitations created by the size, number or accessibility of source populations [3]. If done well, translocations can be an excellent tool for improving the likelihood of a species’ persistence into the future. In numerous cases, such as that of the Californian condor (*Gymnogyps californianus*) and Przewalski’s horse (*Equus ferus*), translocations have been a key measure in preventing species extinction [4, 5].

Two translocation categories have been recognised by the International Union for Conservation of Nature (IUCN); population restoration (movement of fauna within indigenous range) and conservation introductions (movement beyond indigenous range) [2]. Releasing animals outside their indigenous range can be contentious due to the potentially disastrous consequences for existing biota [6]. However, translocating animals within their indigenous range is often difficult as appropriate release sites where threats have been ameliorated are not always available [7]. Conservation introductions have proved useful in overcoming this restriction, creating a more certain future for threatened species that have a distribution so geographically restricted that one single event, such as a fire or a predator incursion, could be catastrophic [8–10].

While commonly attempted, translocations are not always successful [1, 11, 12]. Despite being used over several decades there continues to be a high failure rate, likely exacerbated by these failures often being unreported or unexplained [6, 13]. In addition, numerous translocations result in unknown outcomes, failing to demonstrate a measurable benefit despite the costs incurred. In Australia alone, where more than 35,000 animals have been translocated since the 1970s, 67% of attempts have either failed or resulted in an unknown outcome [14]. This number improves marginally if we only consider conservation introductions, where just under half (47.5%) have not been successful [14]. Given this rate of failure, careful planning needs to be undertaken to confirm that costs incurred attempting translocations do not outweigh the potential benefits of the program [15].

Translocations are expensive, both financially and ecologically [16]. Numerous individuals of threatened species are often the price paid in failed translocations, with additional costs potentially incurred through detrimental impacts to the source populations such as reduced genetic fitness or changes to population dynamics [2, 17]. As such, factors that could influence the outcome of a translocation and their likelihood of occurrence should be thoroughly critiqued and modelled during the planning stages of such a program. This is particularly salient for conservation introductions as there is no precedent for species’ survival at potential release sites [6]. While conservation practitioners intend for translocations to be beneficial, they also need to determine if the action is likely to achieve this aim.

Population viability analysis (PVA) is an effective, transparent and widely used process [18–21] for exploring the long-term impacts of stochastic and deterministic factors on the persistence of populations [22]. Initially developed in the 1980s, PVA became a readily accessible and validated predictive tool in 2000 [23–25]. The predictive power of PVAs designed and informed using expert knowledge, have been shown to be accurate with the risk of population decline closely matching observed outcomes in 21 long-term ecological studies [25].

Despite its current accessibility and predictive accuracy, PVA is a recommended but not required decision-making tool for species translocations [6]. In cases where translocations must be implemented with little notice, managers tend to rely solely on expert knowledge for planning purposes rather than divert resources to also incorporate ecological modelling. Additionally, there exists the perception that ecological modelling cannot be done for data-deficient species and as such there has been contention about the accuracy and role of PVA in predicting extinction probabilities [26]. There is also the misconception that PVAs can only generate results in the form of extinction probabilities and do not provide any useful information on species demographics. However, comprehensive studies have unequivocally advocated the importance and robust nature of PVA in producing unbiased predictions [25, 27, 28].

Here we aim to demonstrate the importance of predictive modelling techniques, particularly PVA for planning and critiquing translocations. We use the example of two species (Djoongari, Shark Bay mouse, *Pseudomys fieldi* and golden bandicoots, *Isoodon auratus barrowensis*) that were both translocated to one island, Doole Island, in the north-west of Western Australia. Djoongari were first translocated to Doole Island in 1993 but despite supplemental translocations spanning an eight-year period, they failed to establish a wild population on the island. In 2011 golden bandicoots were translocated to Doole Island and have since successfully established a persistent population. It should be noted that PVA was not available prior to the first translocation of Djoongari to Doole Island and was not used during the planning stages of the golden bandicoot translocation to Doole Island. We address and critique the criteria used to measure the success of these translocations, provide a retrospective demonstration of predictive modelling in both situations and discuss the demographic factors contributing to the actual outcomes of these conservation introductions.

## Methods and materials

### Translocation site

**Fig 1:**
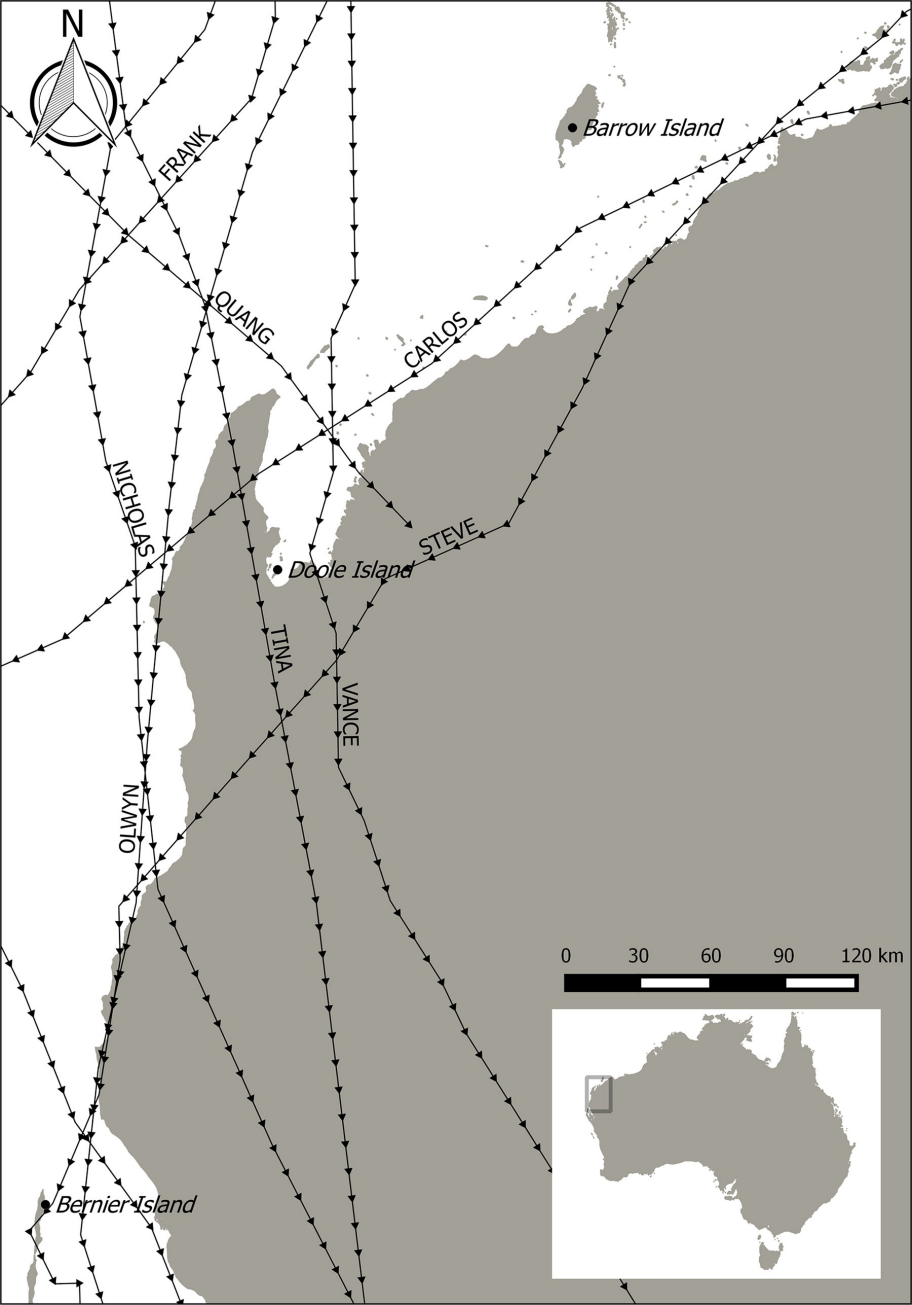
North-west Australia, location of source sites for Djoongari (Bernier Island) and golden bandicoots (Barrow Island) and the release site (Doole Island) for both translocations. Broken lines indicate the paths of severe tropical cyclones that have passed within 100 km of the Exmouth Gulf in the last thirty years (Tina 1990, Frank 1995, Vance 1999, Steve 2000, Nicholas 2008, Carlos 2011, Olwyn 2015, Quang 2015; [32]).

Doole Island (22°27’ S, 114°09’ E), is a nature reserve (Class C; Reserve number 42755) located at the southern end of the Exmouth Gulf, Western Australia (Fig 1). It was chosen as a translocation site for Djoongari and golden bandicoots due to its size (258 ha), ease of access, and similar climate, geology, and habitat types to source population sites (Table 1) [29, 30]. Doole Island was free of any non-indigenous and invasive animal species, no resident species were expected to strongly compete with Djoongari for resources and only one species (*Varanus gouldii*) was expected to compete with golden bandicoots. Despite sub-fossil records of both Djoongari and golden bandicoots from the adjacent mainland [31], there are no records of either species ever having occurred on the island making both translocations conservation introductions as opposed to reintroductions [2].

**Table 1:** Current environmental criteria for identifying potential island translocation sites and descriptions of the source populations of Djoongari (Shark Bay mouse) *Pseudomys fieldi* and golden bandicoot *Isoodon auratus barrowensis* translocations.

Criteria	Doole island	Bernier island	Barrow island
	Translocation site	Source population for Djoongari (Shark Bay mouse)	Source population for golden bandicoot
Island size >100 ha	258 ha	4,150 ha	23,350 ha
Distance from translocation site	-	270 km (south)	210 km (north)
Climate	Arid sub-tropical	Mediterranean	Arid sub-tropical
Geology	Quartzose calcarenite rock, a central claypan and sandstone ridge and coral reef outcrops around the coastline^3^	Sandplain, consolidated dunes, unconsolidated dunes, and travertine rock formation^1^	Limestone overlain by sands, gravels and travertine rock formation^5^
Habitat types	Mixed *Acacia bivenosa* and *Adriana tomentosa* shrubland over herbs and *Triodia epactia* hummock grassland on pink/brown sand. Open *Avicennia marina* mangrove and *Sporobolus virginicus* grassland on coastline. Sparse *Spinifex longifolius* in sandy coastal fringes. *Frankenia pauciflora* and *Tecticornia* sp. dwarf shrubland on limestone rock platform^4^	Flat red soil plateau dominated by *Triodia plurinervata*, and boulder point plateau dominated by *Thryptomene micrantha*, surrounded by steep cliffs, and sandy bays on the east coast dominated by *Spinifex longifolius*^2^.	Western sandy beaches dominated by *Spinifex longifolius*. Inland limestone ridges, plateaus and valleys ranging from hummock grasslands with open low shrubland to pockets of dense shrubs. Dominant species in these areas include *Triodia wiseana* and *T*. *angusta*, *Acacia bivenosa* and *A*. *coriacea*, *Melaleuca* sp. and *Petalostylis* sp.^5^
Exotic vertebrates present	None	None	None
Native competitors present	*Varanus gouldii* (competitor for golden bandicoot only)	Ash-grey mouse (*Pseudomys albocinereus*)	Several species including burrowing bettongs (*Bettongia lesueur*) and brush-tailed possum (*Trichosurus vulpecula*)
Land owner/manager	Crown owned; agency managed	Crown owned; agency managed	Crown owned; industry and agency managed
Accessibility	Easy	Difficult	Moderate
Island use	Nature reserve Class C	Nature reserve Class A	Nature reserve Class A

^1^[33]

^2^[34]

^3^pers. comm M. O’Leary

^4^pers. comm. N. Godfrey

^5^[35].

The habitat of Doole Island is dominated by low open shrubland and *Triodia epactia* grassland with sparse *Spinifex longifolius* in coastal sandy margins (pers. comm N. Godfrey 2017; Table 1). Recent geological surveys state that the island’s highest point is 12.55 m above mean sea level (MSL). High tide level is 1.6 m above MSL, however extensive areas of stormwrack deposits have been found inland of the present shoreline at elevations of up to 5.1 m above MSL (pers. comm M. O’Leary 2017). This low-lying island is located in the most cyclone-prone region of Australia [36] with severe tropical cyclones impacting the island (tracking within 100 km) on average once every two to three years [36] (Fig 1).

### Ethics statement

Translocations to Doole Island were approved by the Western Australian Department of Biodiversity, Conservation and Attractions (and its predecessors) Animal Ethics Committee who are responsible for ensuring that research activities are carried out in accordance with the Australian code for the care and use of animals for scientific purposes. The animal ethics approval number for the translocation of Djoongari and golden bandicoots were CAEEC 11/92 and AEC 2011/13 respectively.

### Translocation one: Djoongari 1993–2001

Once widely distributed from the southwest quadrant of Australia to the arid zones as far north as the Northern Territory and east to New South Wales [37, 38], Djoongari, a robust (30–45 g), long-haired Australian native rodent [39] had declined to only one island population, Bernier Island, by the late 19th century [40]. Located off the west coast of Western Australia, this 4150 ha island was estimated to have a population of between 5000 and 9000 Djoongari, extrapolated from recaptures of Djoongari at five trapping sites [40, 41] (Fig 1). Djoongari are generally associated with coastal vegetation dominated by *S*. *longifolius* and *Olearia axillaris* [42, 43]. Mice form tunnels through this dense coastal vegetation and shelter in burrows dug in the fine sands typically associated with this habitat type. This association between the mice and vegetation has been confirmed by an ecological study that found a positive correlation (r = 0.44) between number of captures and the percentage cover of *S*. *longifolius* as opposed to a negative correlation (r = -0.28) with the percentage cover of *Triodia* sp. (Unpublished data, P. Speldewinde and K. Morris). Unfortunately, no information is available for mainland habitat preferences due to the rapid decline of the species following European settlement [40].

**Fig 2:**
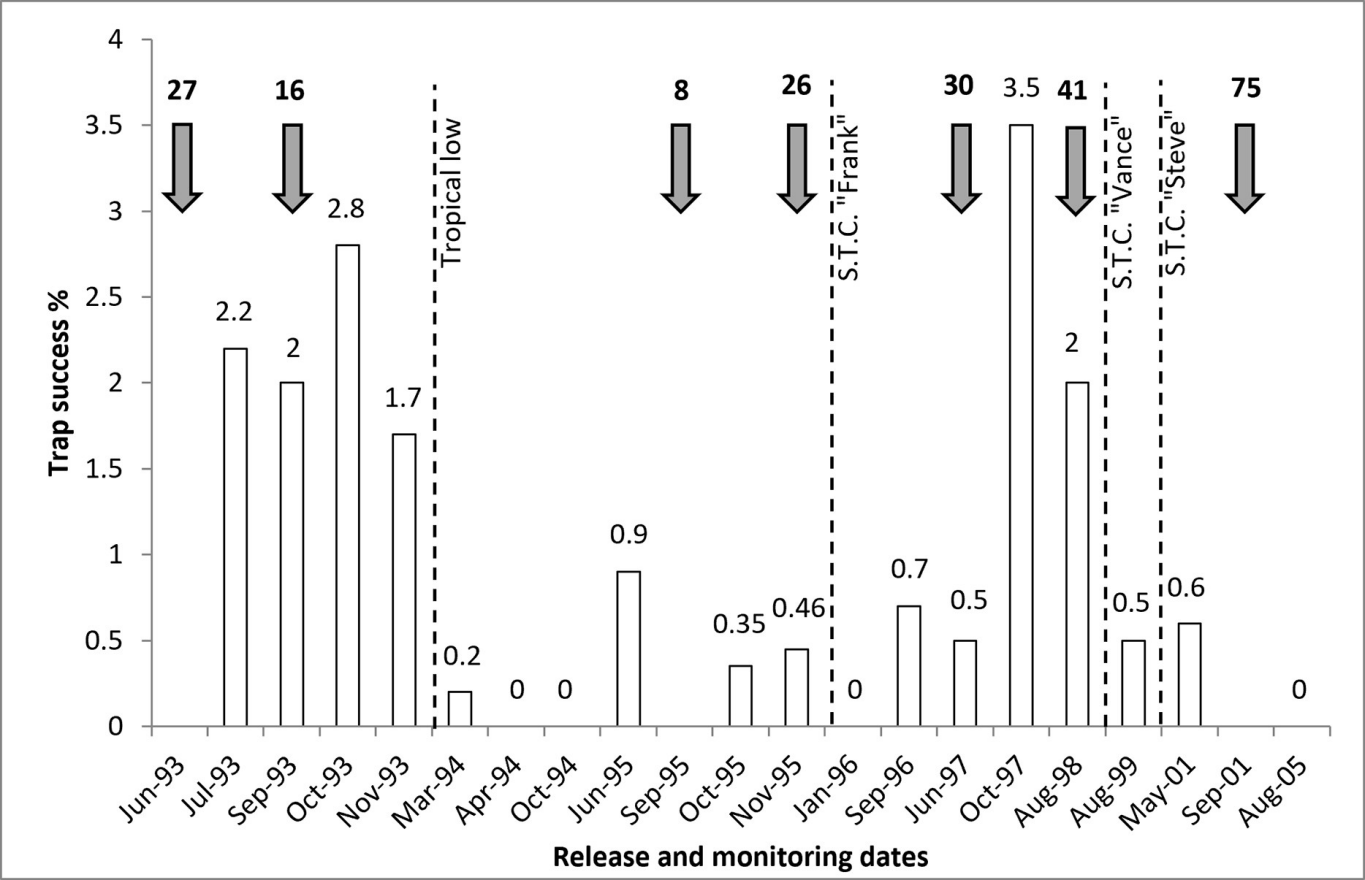
The number of Djoongari released onto Doole Island (shaded arrows) and the associated trap success rates (captures per hundred trap nights) during each monitoring session [45]. Instances of cyclonic events (tropical lows and severe tropical cyclones (S.T.C) impacting the island (passing within 100 km) are indicated by broken lines).

**Fig 3:**
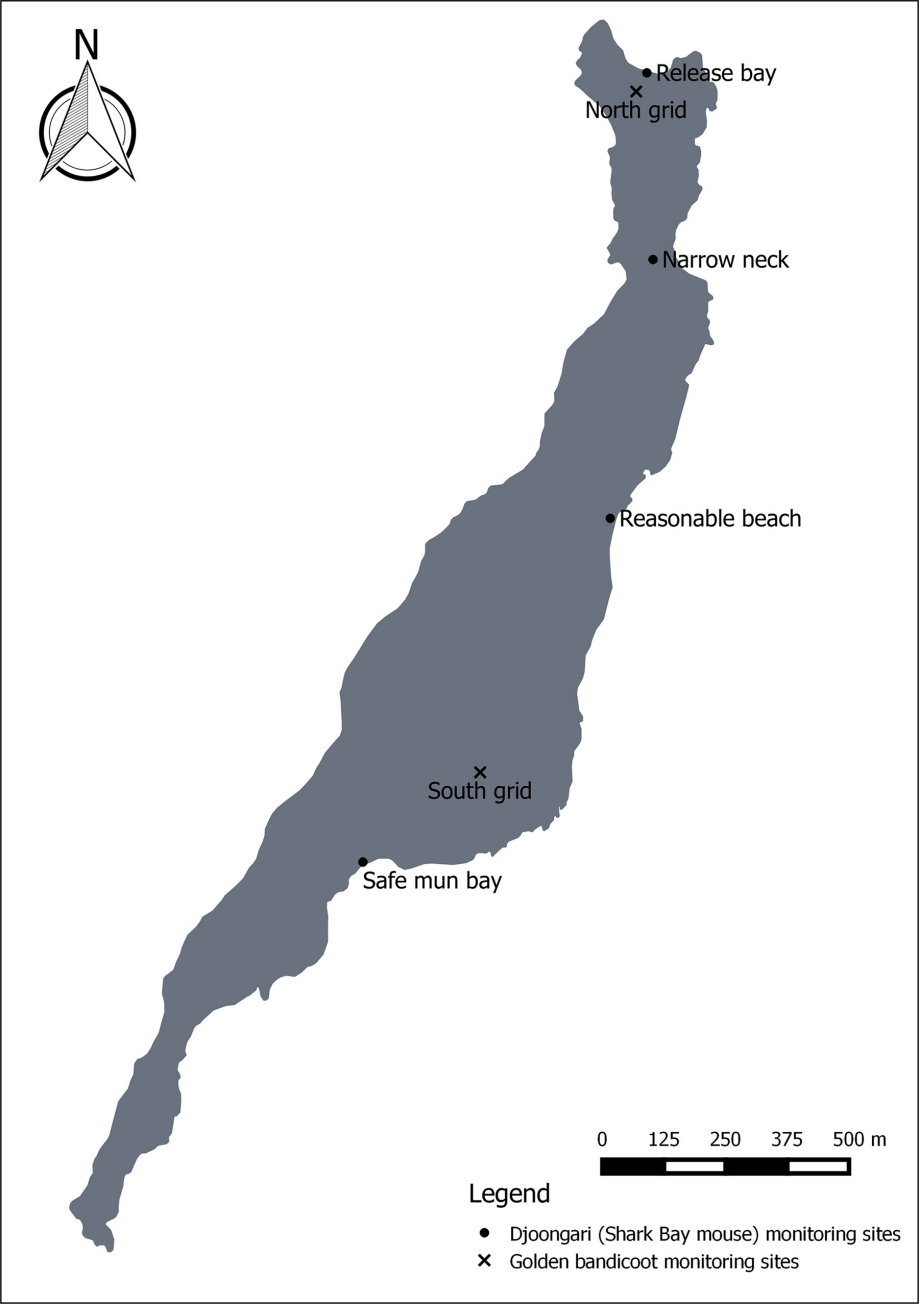
Monitoring sites for Djoongari (Shark Bay mouse) *Pseudomys fieldi* and golden bandicoots *Isoodon auratus barrowensis* on Doole Island, Exmouth Gulf, Western Australia.

Classified as threatened fauna (Endangered) under the 1992 Commonwealth Endangered Species Protection Act, a Djoongari Recovery Plan was drafted that identified the establishment of a self-sustaining population on another offshore island within the recovery criteria [44]. Doole Island was later identified as the translocation site with the success criteria refined to require the population be self-sustaining within five years with demonstrated population growth [40]. Over nine years, seven translocations were undertaken to Doole Island (Table 2; Fig 2). Animals were trapped on Bernier Island using Elliott traps (Elliott Scientific Company, Upwey, Victoria) baited with a mixture of peanut butter, sardines and rolled oats and held individually for up to five days within plastic rodent cages containing a small amount of vegetation and shredded paper. Cages were transferred between the two islands via boat and vehicle, a total travel time of 10.5 hours. Animals were released at the northern end of Doole Island within the vicinity of ‘Release Bay’ (Fig 3). Seventy-eight animals were successfully translocated directly from Bernier Island between 1993 and 1995, while 146 individuals bred at Perth Zoo (stocked from Bernier Island) were released in three separate events between 1997 and 2001.

**Table 2:** Summary of effort and overall outcome for the translocation of Djoongari and golden bandicoots to Doole Island. Outcomes of the translocations were measured against pre-defined criteria for success.

	Djoongari	Golden bandicoot
Translocation	223 individuals in 7 release events spanning 9 years^1^	92 individuals in 1 release event
Criteria for success	A self-sustaining population within 2 years^1^, revised to 5 years^2^.	*Short term (release to 1 year)*
		Survival of 60% of founders
		Recruitment of new individuals
	Densities similar to those on Bernier island^1^, revised to demonstrated population growth^2^	Body weights stable within 6 months of release
		*Medium to long term (1–5 years)*
		Ongoing persistence of the population
		Trap success > 20%
		Continued recruitment
		Population expansion to occupy all
		suitable habitat^3^
Monitoring effort	18 monitoring sessions	6 trapping sessions
	650–1640 trap nights per session	300–400 trap nights per session
	1993–2005	2011–2017
Outcome	Failure	Success

^1^[44]

^2^[40]

^3^[45].

The monitoring of Djoongari post release was completed using radio-telemetry (2 g transmitters on 38 animals between 1993 and 1995), trapping (between 650 and 1640 trap nights per session; Table 2; Fig 2), hair tubes (September 1996) and track observations (noted for all monitoring sessions). Trapping and tracking sites were established on the coastal areas at Release Bay, Narrow Neck, Reasonable Beach and Safe Mun Bay (Fig 3). Monitoring sessions were intended for every six weeks for the first six months, every three months for the next 12 months then annually after 18 months. Actual monitoring followed this prescription initially but was altered in response to the additional translocations (Fig 2). Efforts continued until 2005, culminating in 18 monitoring sessions over a 13-year period (Table 2).

One tropical low (1994) and three severe tropical cyclones (Severe Tropical Cyclone Frank (1995), Severe Tropical Cyclone Vance (1999) and Severe Tropical Cyclone Steve (2000)) passed within 100 km of the Exmouth Gulf during this monitoring period [46] (Fig 1). Vance was the most damaging of these events, intensifying to a category five system that tracked south through the Exmouth Gulf, crossing the mainland coast less than 20 km east of Doole Island. Storm surges exceeding five metres were recorded at Tubridgi Point at the north-eastern extent of the Exmouth Gulf [47], however the exact height at Doole Island is unknown. Visitation to the island five months after Vance revealed that this event destroyed the mangroves on the west coast of the island and vegetation up to 20 m inland of the high-tide mark had been inundated [48], including the coastal habitat that Djoongari are generally associated with [42, 43]. These cyclonic events were suggested as potentially causative in the outcomes of this translocation due to increased exposure to the elements and to threats such as predation following the inundation and destruction of core Djoongari habitat and refuge sites. For that reason the impact of cyclonic events have been included in this analysis.

### Translocation two: Golden bandicoots 2011

The golden bandicoot is the smallest member of the *Isoodon* genus (approximate maximum 670 g), a group of ground dwelling, omnivorous marsupials [49]. Once distributed across more than a third of the Australian mainland, the golden bandicoot has also undergone a drastic range reduction [49]. Now only found in approximately one percent of its former range [50], the golden bandicoot is listed as threatened fauna (Vulnerable) under the Australian Environment Protection and Biodiversity Conservation Act 1999. This species is flexible in its habitat use but is associated with hummock grasslands in arid habitats [49, 51], where animals will shelter under *Triodia* clumps during the day.

**Fig 4:**
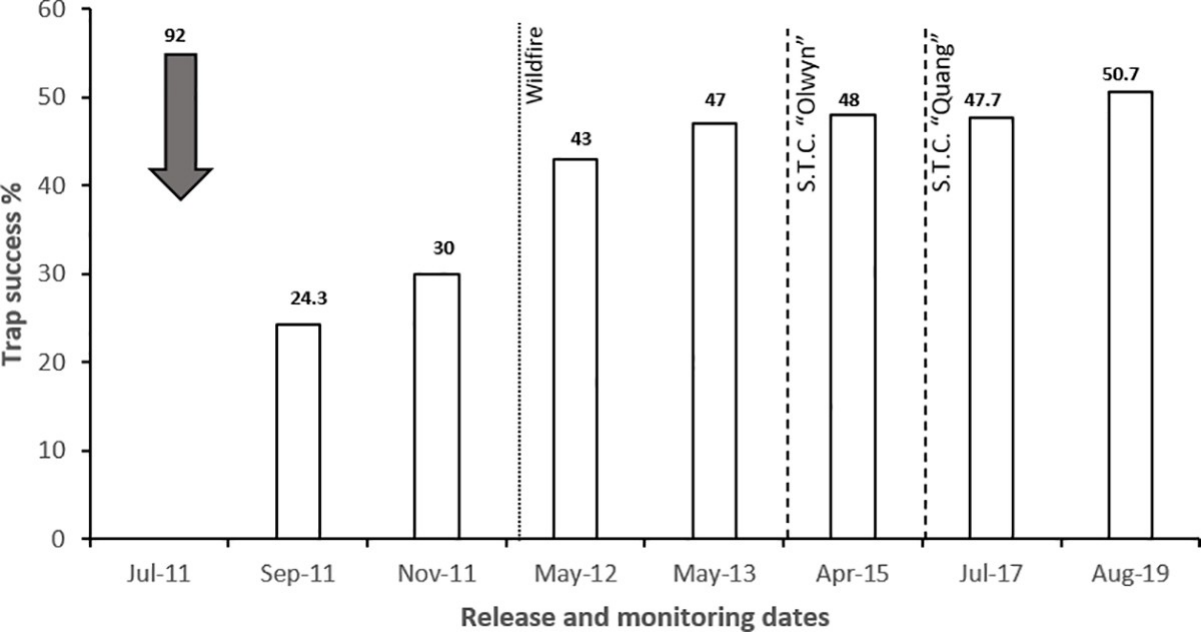
The number of golden bandicoots released onto Doole Island (shaded arrow) and the associated trap success rates (captures per hundred trap-nights) during each monitoring session. Instances of catastrophes during this period are indicated by dotted line (wildfire) and dashed line (severe tropical cyclones (S.T.C) impacting the island (passing within 100 km)).

Translocating golden bandicoots from Barrow Island Nature Reserve (23590 ha) to Doole Island was proposed as a method of ameliorating the impacts from industrial works associated with the Gorgon Gas Development that was taking place on Barrow Island [45]. Barrow Island is one of only two remaining natural populations of the Pilbara subspecies *Isoodon auratus barrowensis*, supporting a population estimated to exceed 20,000 individuals [49]. In July 2011, 92 golden bandicoots were translocated to Doole Island (Fig 4). Animals were trapped on Barrow Island using a combination of Elliott and Sheffield traps (Sheffield Wire Products, Welshpool, Western Australia) baited with a mix of peanut butter, sardines and rolled oats. Golden bandicoots selected for translocation were individually marked with passive integrated transponders (Allflex Australia, Capalaba, Queensland), and then transported in individual black handling bags that were secured within plastic pet carriers with up to three other individuals. These animals were transferred by light aircraft, vehicle and boat, and released at two sites on Doole Island within 24 hours of capture.

Seven criteria for success were defined for this translocation (Table 2). Monitoring of this population to meet these criteria was completed using Elliott trapping (300–400 trap nights per session) and track surveys. Two trapping grids were established, one at the northern end of the island and one in the middle, within close proximity to the release sites (Fig 3). New individuals that were captured were marked using the same method as that in the translocation. Track surveys were undertaken by a team of four people walking the whole island in the period of one day. Teams started at the south end of the island and walked north in a random manner to inspect soft substrate. Monitoring occurred every two months for the first four months, then annually and was reduced to biennial monitoring two years after the release. Total trapping effort of this translocation was 23 days over a seven-year period (Table 2; Fig 4).

Two tropical cyclones occurred during this monitoring period, both in 2015. Severe Tropical Cyclone Olwyn tracked down the western side of the Exmouth Cape as a category three system while Severe Tropical Cyclone Quang travelled across the north of the gulf as a category one system just over six weeks later [52] (Fig 1). The storm surge height for these cyclones was recorded as 3.42 m and 2.62 m respectively, in the town of Exmouth 55 km northwest of Doole Island [53]. While the exact height of the storm surges at Doole Island is unknown, aerial imagery captured after Cyclone Olwyn indicated that as much as 20% of inland *Triodia* sp. vegetation had been destroyed and hence 100% of coastal dune vegetation was likely inundated during this event. In addition to the two cyclones, a wildfire was sparked by lightning in the summer of 2011/2012 and burnt approximately one third of the island’s vegetation.

#### Population estimates

We used open population spatially explicit capture-recapture analysis in the R package ‘openCR’ with the island as a closed survey area [54] to estimate the abundance of golden bandicoots on Doole Island over time. The openCR package applies Pollock’s Robust Design analysis to trapping data with unequal intervals between sessions. Capture-recapture data from three days of translocations during the July 2011 release event and 23 additional trap occasions were entered as 7 primary sessions and 26 secondary occasions with defined time intervals (July 2011, 3 days; September 2011, 4 days; November 2011, 4 days; May 2012, 4 days, May 2013, 4 days, April 2015, 3 days; July 2017, 4 days). We ran models that included capture records from both sexes, and models that only used captures records for a single sex because unpublished field observations suggest sexes have variable behaviour and capture probability. Since our interest was in an estimate of the abundance of bandicoots on Doole Island we predicted the density of bandicoots on Doole Island using the JSSAsecrD model [54], and explored the influence of continuous time and primary sessions on estimates of the detection function intercept (lambda0), the detection function scale (sigma), survival estimates (phi) and density (D) because it was expected that the abundance of a recently translocation population of golden bandicoots would change over time. Models generated by openCR were ranked according to Akaike’s Information Criterion (AIC) [55] and any models with more than one unidentifiable parameter were rejected [54]. We converted the estimates of density and confidence intervals to estimates of abundance by multiplying values by the area of the island (258 ha). We report the estimates of lambda0, sigma, survival and density from the best models (Table 3) in (S1 Table).

**Table 3:** Input parameters for VORTEX simulations for Djoongari and golden bandicoots on Doole Island. Timesteps varied based on the generation time of each species: One timestep for Djoongari is equal to one month; golden bandicoots modelled using four months as a timestep.

	Djoongari		Golden bandicoot	
Parameter	Baseline model*	Parameter variation (min-max)	Baseline model*	Parameter variation (min-max)
Number of iterations	1000	-	1000	-
Number of timesteps	360	-	90	-
Duration of each timestep in days	30	-	120	-
Reproductive System	Polygynous	-	Polygynous	-
First breeding age of females	3^a^	-	1^b^	-
First breeding age of males	3^a^	-	1^b^	-
Maximum age of female reproduction	21^a^	-	15^b^	-
Maximum age of male reproduction	18^a^	-	15^b^	-
Maximum lifespan	36^a^	-	15^b^	-
Carrying capacity	340 ± 65	50–550	500 ± 107	150–950
Maximum number of litters per timestep	1^a^	-	1	-
Average number of progeny per timestep	3.6 ± 1.43^a^	-	2.5 ± 1^b^	-
Maximum number of progeny per timestep	6^a^	-	5	-
Sex ratio at birth in % males	44.5	-	50	-
Males in breeding pool %	100	-	97.8^b^	-
Females reproducing per timestep %	30 ± 20	0–75	70 ± 5	25–100
Annual mortality as a % (SD)				
Adult	10 ± 3	0–20	10 ± 3	0–30
Subadult (Age 2–3)	20 ± 3	0–40	-	-
Juveniles (Age 1–2)	25 ± 10	0–50	-	-
Juveniles (Age 0–1)	25 ± 10	0–50	50 ± 10	25–75
^c^Frequency of catastrophe %				
Cyclonic storm surge	7	-	27	-
Influence of cyclonic storm surge as a proportion of normal values on				
Reproduction	0.5	0–1	0.9	0–1
Survival	0.5	0–1	0.75	0–1
Initial population size	27	-	92	-
Supplementation				
First timestep of supplement	4	-	-	-
Last timestep of supplement	100	-	-	-
Interval between supplements	16	-	-	-
Number of adult females supplemented	17	-	0	-
Number of adult males supplemented	16	-	0	-
Stable age distribution^d^	Yes	-	Yes	-

^a^ [56]

^b^[30]

^c^ frequency of catastrophe is relative to the length of the timestep and is hence equal across species

^d^Real age distribution was not known for either species.

### Population viability analysis

We used Vortex Version 10.1.6.0 [57] for a retrospective PVA of the establishment and persistence of populations of the Djoongari and golden bandicoot populations translocated to Doole Island. Briefly, VORTEX is an individual-based simulation program that models population processes (fecundity, mortality) as discrete, sequential events, with probabilistic outcomes [24, 58]. VORTEX uses random numbers, selected from user-defined distributions that reflect the species demographics, to determine whether each animal in the model lives or dies, and to determine the number of progeny produced by each female each year [24, 58]. Fecundity is assumed to be independent of age after an animal reaches reproductive age. Mortality rates are specified for each pre-reproductive age-sex class and for reproductive-age animals. Population carrying capacity is imposed by a probabilistic truncation of each age class when the population size exceeds the user defined carrying capacity. Environmental variation is incorporated into VORTEX by sampling birth rates, death rates, and the carrying capacity from a user-defined normal distribution. Catastrophes are modelled as sporadic random events that reduce survival and reproduction for one timestep [24, 58]. Users define the probability of a catastrophe occurring and the probable effect of the catastrophe on species survival and reproductive rates (Table 3).

VORTEX requires several inputs describing the demographics of a species. Unfortunately, the demographics of many threatened species are poorly understood, largely due to low numbers of wild animals that may be studied. While research at Perth Zoo has defined some of the demographic parameters for Djoongari in captivity [56], the demographic parameters for Djoongari in the wild are not well known and were even less so at the time of this translocation. Similarly, the demographic parameters for golden bandicoots were ultimately drawn from unpublished data on recently translocated bandicoot populations to Hermite Island, Montebello Archipelago, Western Australia, and Matuwa, in central Western Australia [30]. Due to this uncertainty in demographic parameters, we first built models using the demographic data that was available as a result of the captive breeding program and unpublished research on Bernier Island. We then engaged in a model validation process in which species experts iteratively modified some of the inputs until the results of our baseline models reflected observed abundances at the respective source sites; Djoongari on Bernier Island (unpublished data P. Speldewinde) and golden bandicoots on Barrow Island [60]. The site-specific information in the models that resulted from this process was then substituted with information specific to Doole Island. Finally, we used the sensitivity testing function within VORTEX to systematically vary the inputs used in the PVA for both Djoongari and golden bandicoots (Table 3) and used logistic regression with binomial distribution or linear multivariate analysis to compare variation in inputs to variation in predicted probability of extinction and stochastic growth rate over 30 years, respectively. Ultimately, 2328 parameter combinations were tested in the Djoongari PVA and 1708 combinations in the golden bandicoot PVA.

VORTEX does not allow animals to be born, mature and breed within a fraction of a year. For species with a short generation time, such as Djoongari and golden bandicoots, we need to define a timestep that is less than the generation time. Variation in the duration of a timestep and the number of timesteps included in the PVA for Djoongari (360 timesteps of 30 days) and golden bandicoots (90 timesteps of 120 days; Table 3) varies in accordance with the species biology. Similarly, the frequency of a catastrophe reflects the duration of a timestep for each species rather than actual variation in the frequency of catastrophes.

Habitat availability or island size is a criterion used during the selection of translocation sites, but it is not a parameter within VORTEX. To overcome this disparity, we used carrying capacity, a parameter within VORTEX that describes the approximate maximal abundance for a species within a given area, as a proxy for an assessment of how island size or habitat availability may influence the probability of extinction over 30 years. On Bernier Island, Djoongari have an estimated abundance of 5000–9000 individuals [40]. The average density across all habitats is 1.56 mice/ha, the highest densities of Djoongari (~4.5 mice/ha) are in *Spinifex longifolius* dominated habitat, with lower densities of Djoongari (~1.3 mice/ha) in *Triodia* grasslands (unpublished data, P. Speldewinde).

Doole Island has an area of approximately 258 ha above the high-water mark. Approximately 50 ha of the island consists of unsuitable intertidal or rocky habitats. Another 105 ha consists of *Triodia* grassland, low shrubs, or mangroves. The final 103 ha consists of tussock grasslands, and coastal dune vegetation (Unpublished data; C. Lohr). The sum 208 ha of available habitat could support approximately 324 Djoongari at an average density of 1.56 mice/ha. Alternatively, the sum product of Djoongari density by these two habitat groupings suggest that Doole Island could support up to 600 mice. Due to the high-level of uncertainty in the availability of suitable habitat we varied the carrying capacity for Djoongari in VORTEX from 50–550 mice (Table 3). Being a threatened species, the carrying capacity for golden bandicoots is not well understood, hence we varied the carrying capacity for golden bandicoots on Doole Island from slightly above the current estimate of abundance, 150 to 950 (Table 3).

## Results

### Djoongari translocation

Neither of the two criteria for success against which this translocation was measured were met (Table 2). Despite Djoongari persisting on Doole Island until at least 2001, they could no longer be detected by 2005 (Fig 2). Initial results were good; capture rates remained above two percent for the six months immediately following the first release, captured animals were always in a healthy condition and 16 young born on the island were detected following release. Radio tracking in combination with hair tubes and track observations indicated that animals were predominantly using the coastal *S*. *longifolius* habitat and regularly sheltering in the hollows of mangroves (*Avicennia marina*). Radio tracking also revealed some mortalities; two animals in 1993 and later three in 1995 were consumed by the resident goanna species *Varanus gouldii*.

From 1994, capture rates dropped to be consistently below one percent, with zero captures in four sessions. The only exceptions were two trapping sessions in 1997 when trap success reached two and 3.5% (Fig 2). However, these sessions followed large releases of captive bred animals and only one third of these captures featured Djoongari born on Doole Island. While Djoongari persisted in low numbers in following years, no population growth was evident and only three and four animals were captured in 1999 and 2001 respectively. An analysis of the capture-recapture records was not attempted for this species due to limited data.

### Golden bandicoot translocation

Of the three pre-defined ‘short term’ criteria for success for the golden bandicoot translocation, only one was met within the designated time-frame (Table 2). Twelve months following release, only 40.2% of founders were recaptured. Fifty-two new golden bandicoots were captured during the three monitoring sessions in the same period. Animals gained weight in the first twelve months, with the weight of males increasing to a maximum average of 39.2% heavier than the release weight, before declining in late-2012 and stabilising at approximately 8.65% more than the average release weight.

All four ‘medium to long-term’ criteria for success were met in this translocation. The population continues to persist with a trap success rate of 47.7% achieved in the most recent monitoring session of 2017 (Fig 4). This session also included the capture of 83 new individuals indicating that recruitment has continued. Results from the best open CR model (Table 3) suggest that the survival rate within the sampled population was 99% (S1 Table). Bandicoots had dispersed from the release sites to occupy the whole island by mid-2012. Following the wildfire in 2012, tracks and diggings were clearly evident across the island suggesting the fire had minimal impact on the bandicoot activity.

**Fig 5:**
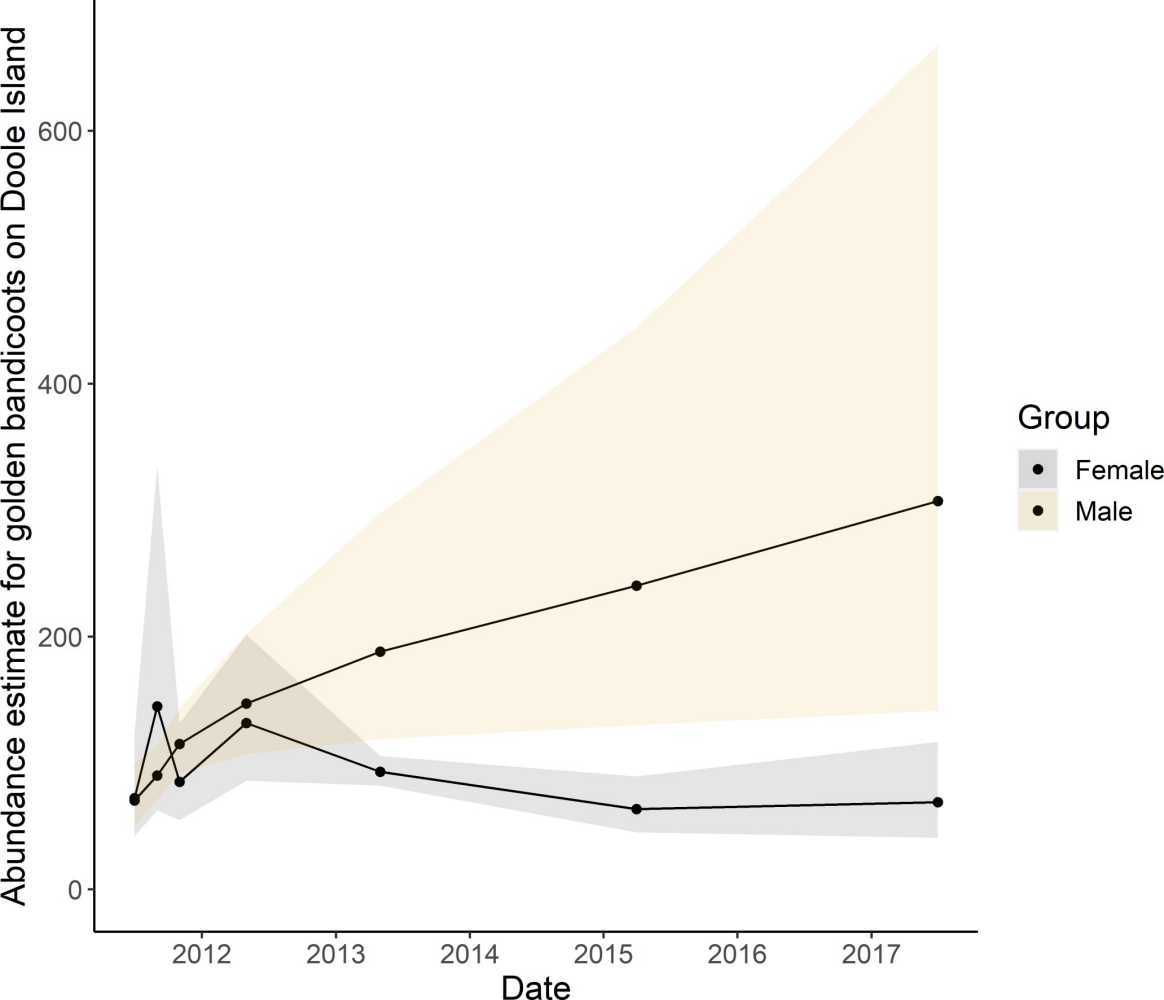
Real abundance estimates (density estimates multiplied by 258 ha) and 95% confidence interval for the female and male golden bandicoot population sampled by two trapping grids on Doole island, Western Australia from the best sex-specific models generated by open population spatially explicit capture-recapture analysis, model type JSSAsecrD.

Analysis of capture-recapture data for Doole Island golden bandicoots via openCR suggests that the abundance of golden bandicoots on the island has increased over time with a slowing rate of increase, (Fig 5). We estimated the abundance of male and female bandicoots separately (Model 1 and 2, Table 4). Males appear more abundant than females, but the density estimates had very wide 95% confidence intervals (S1 Table) suggesting unmodelled transience or heterogeneity in the detectability of individuals [61]. Female abundance appears to have fluctuated in the 12 months post-translocation but then stabillised at approximately 100 individuals. The best models for predicting bandicoot abundance were single sex models with primary session as a covariate in estimates of lambda0, sigma, and density for females, and secondary sessions as a covariate in estimates of lambda0, sigma, and density for males (Table 4, S1 Table). The probability of survival was near identical for the two sexes (females = 99.88%, males = 99.89%) and did not vary significantly with time.

**Table 4:** Ranked models generated by open population spatially explicit capture-recapture analysis, model type JSSAsecrD for golden bandicoots on Doole Island, Western Australia.

Model	Group	AIC	Delta AIC	Model Likelihood	Number Parameters
lambda0~t phi~1 D~t sigma~t	Female only	5003.70	0	-2479.85	22
lambda0~Session phi~1 D~Session sigma~Session	Male only	7538.64	2534.94	-3762.32	7
lambda0~Session phi~1 D~Session sigma~Session	Both sexes	12640.80	7637.10	-6313.40	7
lambda0~t phi~1 D~1 sigma~1	Both sexes	12749.19	7745.49	-6364.60	10
lambda0~1 phi~1 D~1 sigma~t	Both sexes	12813.29	7809.58	-6396.64	10
lambda0~1 phi~t D~1 sigma~1	Both sexes	12822.83	7819.13	-6402.41	9
lambda0~1 phi~1 D~1 sigma~1	Both sexes	12824.92	7821.22	-6408.46	4
lambda0~1 phi~1 D~t sigma~1	Both sexes	12825.25	7821.55	-6402.62	10

Phi = apparent survival; lambda0 = detection function intercept; sigma = detection function scale (m); D = density per hectare; t = primary session; Session = secondary session or continuous time; (~1) = parameter held constant. Separate models run for each sex. Unidentifiable models with a low rank were removed from the final model set [59]. Weak models have been included to demonstrate the variation in explanatory power.

### Population viability analysis

**Fig 6:**
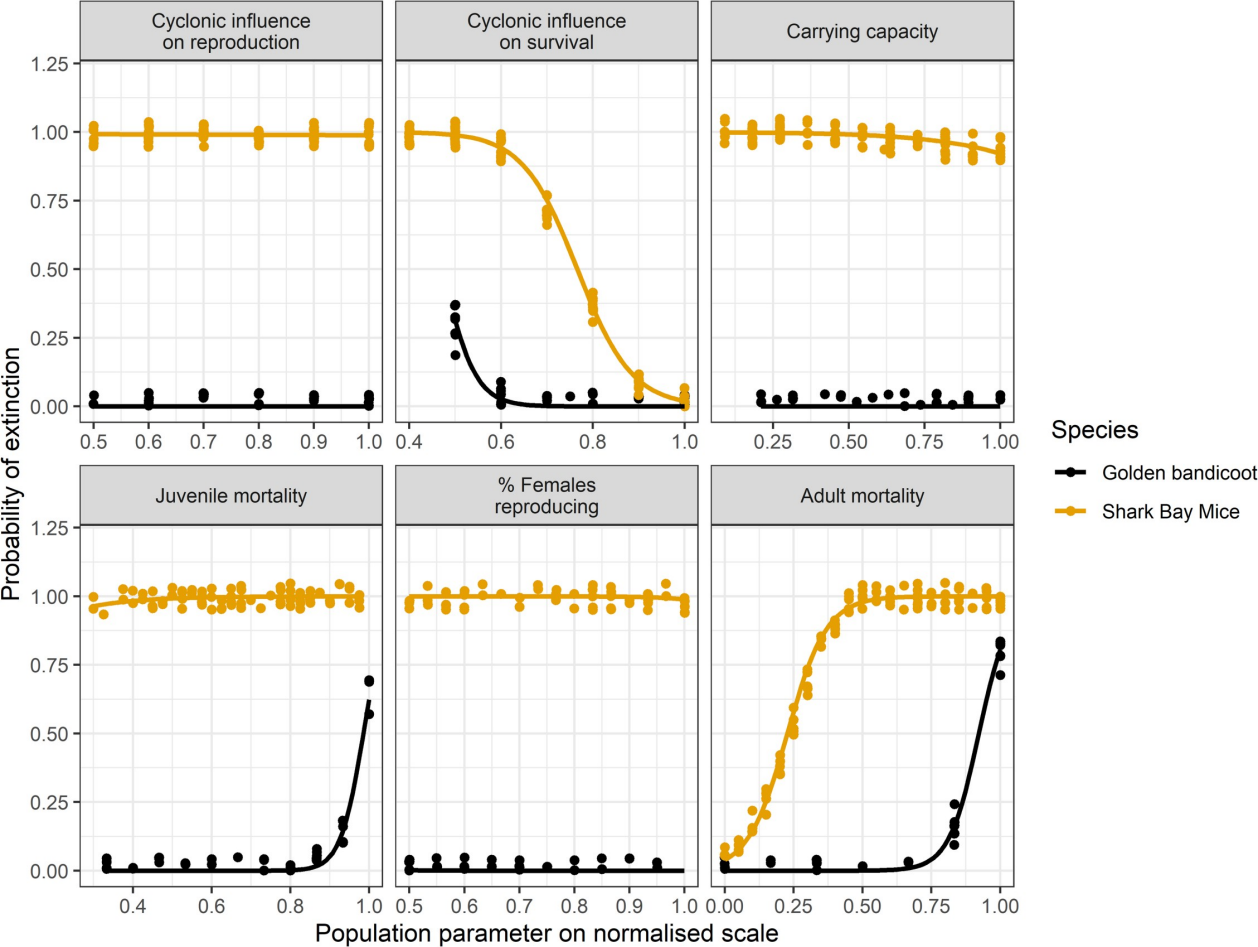
Logistic regression with binomial distribution of variation in population parameters for Djoongari (Shark Bay mouse) and golden bandicoots against the probability of extinction estimated in VORTEX. Catastrophe reduce survival = CS = Proportional reduction in original survival rates due to a catastrophe; Catastrophe reduce reproduction = CR = Proportional reduction in original percentage of females reproducing per timestep due to a catastrophe.

The results of the PVA sensitivity testing suggested that inputs for Djoongari and golden bandicoot mortality and reproduction rates were the most influential parameters on the probability of Djoongari and golden bandicoot extinction over 30 years (Table 5 and 6; Fig 6). For Djoongari there were significant interactions between the percentage of females reproducing per timestep and the proportional reduction in survival rates due to a catastrophe. Similarly, for golden bandicoots the proportional reduction in original survival rates due to a catastrophe significantly reduced survival and reproduction rates (Table 6). In VORTEX the impact of a catastrophe is a multiplier of natural mortality and reproductive rates [56] and hence augmentation of the impact of natural mortality and reproduction rates on the probability of extinction is expected.

**Table 5:** Results of binomial logistic regression generated by VORTEX sensitivity testing of five demographic parameters for Djoongari.

Parameter	Estimate	Std. error	z-value	P-value
MA	5.13E-01	4.21^−2^	12.19	<0.001**
CS	1.22	1.00	1.22	0.22
R	2.08^−1^	3.01^−2^	6.90	<0.001**
CR	-6.07^−1^	4.67^−1^	-1.3	0.19
K	-1.80^−3^	1.60^−3^	-1.12	0.26
MJ(2–3)	4.15^−2^	1.12^−2^	3.70	<0.001**
MJ(1–2)	4.28^−2^	9.19^−3^	4.66	<0.001**
MJ(0–1)	5.03^−2^	7.27^−3^	6.92	<0.001**
CS:R	-4.81^−1^	5.28^−2^	-9.12	<0.001**
R:K	-2.15^−4^	8.59^−5^	-2.50	0.012*

MA = Mortality of adults; CS = Proportional reduction in original survival rates due to a catastrophe; R = Percentage of females reproducing per timestep; CR = Proportional reduction in original percentage of females reproducing per timestep due to a catastrophe; K = Carrying capacity; MJ(*x*) = Mortality of juveniles (x = age class).

* Significant parameter p<0.05

** Significant parameter p≤0.01.

**Table 6:** Results of binomial logistic regression generated by VORTEX sensitivity testing of five demographic parameters for golden bandicoots.

Parameter	Estimate	Std. error	z-value	P-value
MA	-0.07	0.18	-0.40	0.69
CS	-6.68	6.15	-1.09	0.28
R	0.17	0.06	2.65	0.01**
CR	18.20	4.79	3.80	0.01**
K	7.38^−3^	5.44^−3^	1.36	0.18
MJ(0–1)	-0.32	0.10	-3.17	0.01**
MA:CS	0.58	0.14	4.20	0.01**
MA:R	7.68^−4^	1.27^−3^	0.61	0.55
MA:CR	0.09	0.14	0.64	0.53
MA:K	2.09^−4^	1.48^−4^	1.42	0.16
MA:MJ(0–1)	-1.73^−3^	1.40^−3^	-1.24	0.22
CS:R	-0.35	0.08	-4.30	0.01**
CS:CR	-17.90	6.54	-2.73	0.01**
CS:K	-0.01	6.75^−3^	-1.93	0.06.
CS:MJ(0–1)	0.36	0.08	4.47	0.01**
R:CR	-0.23	0.06	-3.62	0.01**
R:K	-1.55^−4^	6.40^−5^	-2.42	0.02*
R:MJ(0–1)	2.67^−3^	7.53^−4^	3.55	0.01**
CR:K	-5.36^−3^	4.09^−3^	-1.31	0.2
CR:MJ(0–1)	0.13	0.08	1.55	0.13
K:MJ(0–1)	1.95^−4^	9.42^−5^	2.07	0.04*

MA = Mortality of adults; CS = Proportional reduction in original survival rates due to a catastrophe; R = Percentage of females reproducing per timestep; CR = Proportional reduction in original percentage of females reproducing per timestep due to a catastrophe; K = Carrying capacity; MJ(*x*) = Mortality of juveniles (x = age class).

* Significant parameter p<0.05

** Significant parameter p≤0.01.

Interestingly, the inflection points of the binomial regressions suggested that Djoongari are a less robust species. For example, the inflection point for the percentage of female Djoongari reproducing was 52.6% whereas for golden bandicoots it was 35.5%. If fewer females reproduced per timestep than these thresholds, then the population was inclined towards extinction. Similarly, if the mortality rate for adult Djoongari exceeded 4.5% the population was inclined towards extinction whereas golden bandicoots could withstand an adult mortality rate of 27.5% (Fig 6). The baseline value for adult mortality in Djoongari was 10% (Table 3). If a catastrophe further reduced Djoongari survival by 50% then we could expect a high probability of extinction.

**Fig 7:**
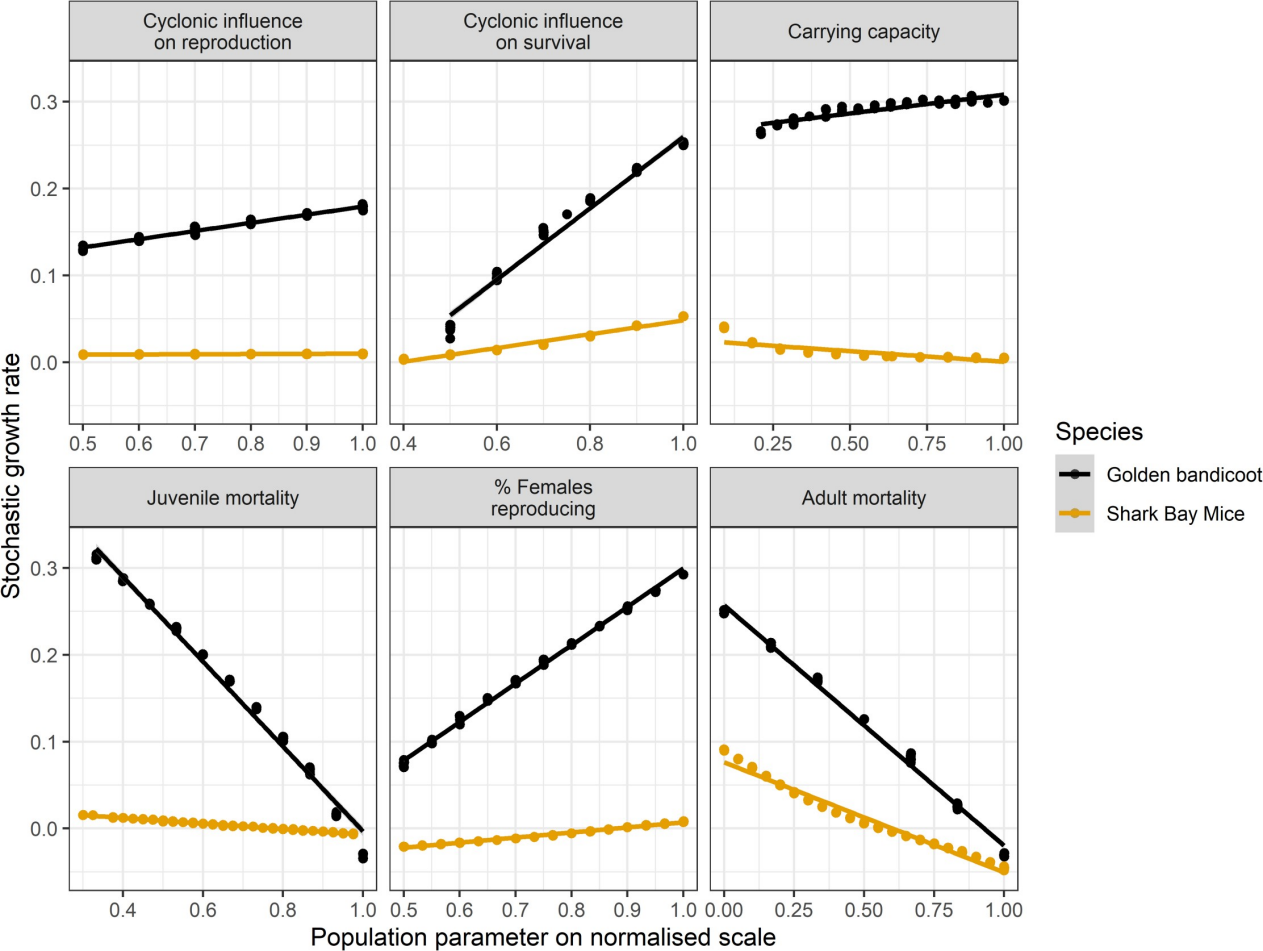
Linear regression of variation in population parameters for Djoongari (Shark Bay mouse) and golden bandicoots against the stochastic growth rate estimated in VORTEX. Catastrophe reduce survival = CS = Proportional reduction in original survival rates due to a catastrophe; Catastrophe reduce reproduction = CR = Proportional reduction in original percentage of females reproducing per timestep due to a catastrophe.

Analysis of the stochastic growth rate of Djoongari and golden bandicoots also suggested that golden bandicoots are a more robust species with greater demographic plasticity. When the stochastic growth rate is less than zero species abundance will decline. Baseline values used in the Djoongari PVA (Table 3) resulted in stochastic growth rates very close to zero whereas demographic parameters for golden bandicoots had to be pushed beyond the range of reasonable uncertainty before stochastic growth rates dipped to zero: for example the percentage of females reproducing per timestep had to drop from a baseline of 70% to 30% (Fig 7).

## Discussion

### Djoongari

The translocation of Djoongari to Doole Island was unsuccessful: this conclusion has been drawn from the failure of the population to meet criteria for success during the project period and has been confirmed by nil detections of Djoongari throughout the golden bandicoot translocation program. This was also the outcome predicted by the retrospective PVA, however there is some discrepancy between the predicted causes of failure and the actual events that occurred.

The frequency and impact of catastrophes, in this instance, storm surges resulting from tropical lows and severe tropical cyclones was a significant explanatory variable in the PVA (Table 5; Fig 6). This factor was included in the PVA as it had been suggested as playing a causative role in the failure of this translocation: Doole Island resides at the bottom of the Exmouth Gulf, an area that is shallow and sharply concave in shape–two features conducive to high storm surges [62]. Storm surges were likely problematic for Djoongari as they predominantly used burrows under the coastal *Spinifex longifolius* and hollows in *Avicennia marina* as refuges, which were inundated and washed away in these surge events. However, it is unlikely that storm surges were the sole cause of failure in the Djoongari translocation, as no cyclonic events or storm surges occurred between the final release in 2001 and the final monitoring session in 2005.

The susceptibility of Djoongari to predation was demonstrated repeatedly when radio-collared individuals were consumed by *Varanus gouldii*. Goannas also preyed upon translocated Djoongari on North West Island in the Montebello Archipelago, approximately 270 km northeast of Doole Island [48]. Whereas wild mice translocated from Bernier Island to Doole Island would not have been naïve to *V*. *gouldii*, which occurs on both islands [42], Djoongari released on North West Island were captive bred and more likely to be predator naïve. Within ten days, 30% of radio-collared Djoongari released on North West Island had been consumed by *V*. *gouldii* [48]. Yet, this translocation program was so successful within twelve years the population on North West Island was first used as a source for further translocations [63] suggesting that predation is also not the sole cause of the failure on Doole Island. Approximately 83 hectares of the vegetation on North West Island is dense *S*. *longifolius* as compared to 20 hectares of sparse *S*. *longifolius* on Doole Island. The presence of dense *S*. *longifolius* on fine substrate suitable for burrow refuges may provide shelter against native predators and subsequently reduce mortality rates for Djoongari.

Our analysis of the influence of carrying capacity on species persistence suggested that it was an insignificant sole variable (Tables 5 and 6). However, Doole Island is dominated by *Triodia* grassland and open shrubland with very little dune habitat featuring soft sands with dense *S*. *longifolius*. Trapping and radio-tracking of Djoongari on Bernier Island found that individuals captured in *Triodia* still utilised the *S*. *longifolius* habitat, sometimes moving up to a kilometre to do so [41, 64]. This indicates that while Djoongari can use *Triodia* habitat, they may not be able to persist long term without access to high quality *S*. *longifolius*. As animals captured on Doole Island were always in a healthy condition, it is unlikely that this utilisation of *S*. *longifolius* is food related. Rather large dense clumps of *S*. *longifolius* may be necessary habitat for this species and the *Triodia* grasslands that dominate Doole Island may be suboptimal habitat for avoiding predation and providing breeding sites.

The presence of sufficient, quality habitat is important to the survivorship of this species beyond providing refuges from predators. Whilst in captivity, intraspecific aggression was witnessed in overcrowded situations to the point of death on some occasions and cannibalism in others [56]. Several of these deaths included juveniles that were either killed by parents or other littermates. If habitat and burrows were limiting on Doole Island, overcrowding may have resulted–a significant factor given the influence that mortality of both adults and juveniles had on the stochastic growth rate predicted by VORTEX. The percentage of females reproducing also had a notable influence on the stochastic growth rate for this species and at least 25% of females in the population needed to reproduce in each timestep for a positive growth rate to occur. Habitat availability is again relevant here due to the requirement for burrows to reproduce but also as females are prone to aborting young in stressful situations [65]–situations that may result from overcrowding and intraspecific aggression.

PVA was unavailable prior to the first translocation of Djoongari to Doole Island and could not have been used in the decision-making process for this translocation, however it has been useful in resolving our understanding of why this translocation failed. The outputs of our model, particularly the plasticity in stochastic growth rates and extinction probabilities, directed us to look at factors that had not been considered before, demonstrating how these approaches can be used (1) to inform decision making when appropriate information is available and (2) identify where crucial information is lacking. Despite the discrepancy between the cause of failure predicted by the PVA (influence of catastrophe) and the actual events, we still advocate for the use of PVA in these situations as the outputs of the model (stochastic growth rates) did direct us to look at factors that had not been considered before. Further, given what we have learned about this failure we advocate for the quantification of likely refuges at proposed translocation sites and concurrent assessments of critical habitat and population viability in recovery plans [66, 67] as well as the impact of natural disasters to be explicitly considered in the planning of translocations.

### Golden bandicoots

The translocation of golden bandicoots to Doole Island was successful as all medium to long-term criteria for success were met in this period. Persistence of golden bandicoots on Doole Island was also predicted using the retrospective PVA. We contend that this is due to their capacity to utilise the inland hummock grassland and shrubland as habitat. The use of *Triodia* as refuge sites by this species has been demonstrated on Marchinbar Island in the Northern Territory [51], as has their persistence in co-existence with native predators on Barrow Island [34]. The hummock grasslands of Doole Island do become inundated during storm surges but as opposed to the coastal vegetation favoured by the Djoongari, are largely retained following these events. Based upon tracks and scats, bandicoots on Doole Island do use the coastal areas but also have the capacity to retreat and capitalise on the inland habitat. This mobility and habitat flexibility has been demonstrated in their persistence following two cyclonic events (Severe Tropical Cyclones Olwyn and Quang) and a wildfire since the translocation to Doole Island.

While all medium to long-term criteria for success were met for this translocation, two of the three short-term criteria were not. This was clearly not due to the failure of the translocation but due to the criteria themselves. For instance, the criteria for stable body weights within six months was not met as weights did not begin to stabilise until 24 months after release. This criterion is quite ambiguous and could have technically been achieved if all animals lost condition following the translocation and maintained unhealthy body weights. Specific language with quantitative thresholds such as ‘maintain weights equal to or above average weight of source population’ would be more appropriate.

Only 40.2% of the founding golden bandicoots were recaptured suggesting that the second criteria for success which was the survival of 60% of founders following the release was also not met. Results from the openCR model, however, suggest that on average 99% of the sampled population survived between trapping sessions. Two factors contribute to these contrasting results. Firstly, the two trapping grids implemented to monitor golden bandicoots covered approximately 3% of Doole Island; an area that may be extended up to 20% of the island if we assume all residents within a 300 m buffer of the grids may be captured. Therefore the sampling effort may have been insufficient to capture 60% of the founders, particularly given the high dispersal distance that was expected and observed (i.e. an animal released at ‘Release Bay’ were recaptured two months later at the southern trapping grid approximately two kilometres further south). Secondly, the recruitment of new individuals would have limited the number of traps available for founder animals. Given that trapping was the only technique used monitor two criteria for success (i.e., capture of 60% of founders, and the recruitment of young), these two criteria were forced to compete for data, with the success of one having the potential to cancel out the success of the other. Other methods such as radio-tracking should have been used to confirm survivorship of founders. Alternatively the criteria themselves could have been altered to recognise the difference between the desired short term survival of founders and the desired long-term survival of the whole population.

These examples highlight the need for translocation criteria for success to be quantifiable and situation-specific. Further, they need to acknowledge proposed methods of data collection, confounding factors amongst criteria, and ideally a power-analysis identifying the quantity of monitoring required to collect sufficient data to address the criteria. Failing to capture the true status of a translocation could result in the unnecessary use of resources or even implementation of actions (e.g. exit strategies) that could be detrimental to achieving the intended outcome of the program [2].

## Lessons for the future

Closed systems such as islands and fenced enclosures that are free of introduced predators are popular options for translocations as they have a low predator invasion or re-invasion risk as compared to open systems [68–70]. Islands in particular are highly appealing as release sites compared to their mainland counterparts where ongoing costs to maintain a predator-free environment are much higher [71]. Despite the potential advantages and relative success of islands as release sites over open systems [72], consideration still needs to be given to all potentially threatening processes; an island free of introduced predators does not guarantee success. We have demonstrated that the effects of localised catastrophes, habitat restrictions and native predators should have been considered within selection criteria for release sites. Similarly, management agencies should consider the overall robustness of a species to environmental variation prior to investing in translocation programs. Had predictive modelling such as PVA been available and the process to inform such a model been undertaken prior to the translocation of Djoongari, perhaps the funds (~$740,000 AUD including monitoring the source population, translocation and monitoring the Doole Island animals and costs of captive breeding [40]), resources and animals used in this attempt could have been directed elsewhere for greater benefit. PVAs do not replace or discount the value of expert knowledge, they are another tool that can help improve outcomes if implemented within their limitations, informed by those with expert knowledge and interpreted by those who understand the context of the data being produced. Strategic decision making processes that involve predictive modelling and sensitivity analysis are particularly important for conservation introductions, but we suggest that the importance is also valid for reintroductions given that these release sites have often been physically, functionally or climatically altered between extirpation and reintroduction of species.

As suggested by multiple other authors, translocations and the criteria to assess their success need to be designed on a case by case basis. Critical planning that takes into account the complex array of interactions that could determine success must be undertaken specifically for each scenario [73–75]. By using predictive modelling, consideration is given to a suite of influencing factors and how they will interact to determine the outcome of the translocation attempt. We also argue this same consideration of specificity and interacting factors be recognised when defining criteria for success. If these criteria form milestones and inform future management actions, ambiguity or discrepancy could mean that results incorrectly represent the situation. The consequences of ignoring these factors have the potential to be extremely detrimental as overall cost, time and effort, in addition to numerous individuals of threatened species, could potentially be directed towards efforts that are more likely to result in successful outcomes.

## Supporting information

S1 Table Estimates of lambda0, sigma, survival and density from the best models generated by openCR for the abundance of golden bandicoots on Doole Island. (XLSX)
